# Supplementary material for: Inadequate Energy Delivery Is Frequent among COVID-19 Patients Requiring ECMO Support and Associated with Increased ICU Mortality
Source: Nutrients. 2023 Apr 27;15(9):2098. doi: 10.3390/nu15092098 (PMC10181417; doi:10.3390/nu15092098)
Supplement: Supplementary file 1 [file nutrients-15-02098-s001.zip › nutrients-2362385-supplementary.pdf]

Supplemental file

Inadequate Energy Delivery Is Frequent among COVID-19 Patients Requiring ECMO Support and Associated with Increased ICU Mortality

Mathias Schneeweiss-Gleixner [1,*], Bernhard Scheiner [1], Georg Semmler [1], Mathias Maleczek [2,3], Daniel Laxar [2,3], Marlene Hintersteininger [1], Martina Hermann [2,3], Alexander Hermann [4], Nina Buchtele [4], Eva Schaden [2,3], Thomas Staudinger [4] and Christian Zauner [1]

[1] Department of Medicine III, Clinical Division of Gastroenterology and Hepatology, Medical University of Vienna, 1090 Vienna, Austria
[2] Department of Anesthesia, Intensive Care Medicine and Pain Medicine, Medical University of Vienna, 1090 Vienna, Austria
[3] Ludwig Boltzmann Institute for Digital Health and Patient Safety, Medical University of Vienna, 1090 Vienna, Austria
[4] Intensive Care Unit 13i2, Department of Medicine I, Medical University of Vienna, 1090 Vienna, Austria
* Correspondence: mathias.schneeweiss@meduniwien.ac.at; Tel./Fax: +43-1-40400-47970

Supplemental Tables

Supplementary Table S1: Comorbidities according to ICU mortality.

Comorbidities	Overall	ICU Surv.	ICU Non-Surv.	p=
**Arterial hypertension, No. (%)**	59 (57.8	32 (53.3)	27 (64.3)	0.3121
**Obesity, No. (%)**	48 (47.1)	28 (46.7)	20 (47.6)	>0.9999
**Diabetes mellitus, No. (%)**	26 (25.5)	14 (23.3)	12 (28.6)	0.6458
**Cardiovascular disease, No. (%)**	6 (5.9)	1 (1.7)	5 (11.9)	0.0790
**Underlying pumonary disease, No. (%)**	11 (10.8)	4 (6.7)	7 (16.7)	0.1926
**Immunosuppression, No. (%)**	1 (1.0)	1 (1.7)	0 (0)	>0.9999
**Chronic kidney disease, No. (%)**	2 (2.0)	1 (1.7)	1 (2.4)	>0.9999
**No underlying disease, No. (%)**	21 (20.6)	15 (25.0)	6 (14.3)	0.2210

Abbreviations: Surv., survivors; Non-Surv., non-survivors; No., number; %, percent.

Supplementary Table S2: Changes in nutrition support over the course of ECMO therapy.

Nutrition data	Overall	ICU Surv.	ICU Non-Surv.	p=
**Daily calorie del. Day 1-3 (% of requ.), mean±Std.**	63.93 (26.64)	63.60 (27.39)	64.40 (25.63)	0.7963
**Daily calorie del. Day 1-7 (% of requ.), mean±Std.**	67.27 (26.92)	68.40 (26.74)	65.64 (27.14)	0.1852
**Daily calorie del. Day 8-14 (% of requ.), mean±Std.**	75.07 (28.53)	75.27 (28.53)	74.78 (28.60)	0.8451
**Daily protein del. Day 1-3 (g/kg BW/d), mean±Std.**	0.62 (0.39)	0.61 (0.40)	0.63 (0.33)	0.5420
**Daily protein del. Day 1-7 (g/kg BW/d), mean±Std.**	0.65 (0.37)	0.66 (0.37)	0.65 (0.37)	0.9315
**Daily protein del. Day 8-14 (g/kg BW/d), mean±Std.**	0.74 (0.35)	0.72 (0.35)	0.76 (0.35)	0.2724

Abbreviations: ICU, intensive care unit; Surv., survivors; Non-Surv., non-survivors; del., delivery; requ., requirements; Std., standard deviation; g, gram; kg, kilogram; BW, actual body weight; d, day.

Supplementary Table S3: Basic characteristics according to adequacy of energy delivery.

Basic Characteristics	overall	mean kcal delivery ≥ 70%	mean kcal delivery < 70%	p=
**Number of Patients, No. (%)**	102 (100)	41 (40.2)	61 (59.8)	
**Age (years), median (IQR)**	57 (50-62)	57 (50-63)	57 (51-62)	0.6694
**Male, No. (%)**	73 (71.6)	29 (70.7)	44 (72.1)	>0.9999
**Weight (kg), median (IQR)**	90 (80-100)	80 (74-98)	95 (85-110)	**0.0018**
**BMI (kg/m^2), median (IQR)**	29 (26-35)	28 (24-33)	30 (27-36)	0.0654
**SOFA at admission, median (IQR)**	8 (7-9)	7 (7-9)	8 (7-9)	0.8476
**SOFA at ECMO Start, median (IQR)**	8 (7-9)	8 (7-9)	8 (7-10)	0.9601
**SAPSII at admission, median (IQR)**	42 (37-49)	42 (34-50)	42 (37-50)	0.9444
**SAPSII at ECMO Start, median (IQR)**	40 (34-46)	40 (34-49)	40 (34-46)	0.9526
**ECMO duration (days), median (IQR)**	20 (11-31)	26 (15-46)	14 (9-22)	**<0.0001**
**ICU LOS (days), median (IQR)**	35 (22-57)	51 (30-72)	27 (19-45)	**<0.0001**

Abbreviations: kcal, kilocalories; %, percent; No., number; %, percent; IQR, interquartile range; kg, kilogram; m^2, square meter; BMI, body mass index; SOFA, Sequential Organ Failure Assessment Score; SAPSII, Simplified Acute Physiology Score II; ECMO, extracorporeal membrane oxygenation; ICU, intensive care unit; LOS, length of stay.

Supplementary Table S4: Daily energy/protein delivery over the course of ECMO therapy according to adequacy of energy delivery.

Nutrition data	Overall	mean kcal delivery ≥ 70%	mean kcal delivery < 70%	p=
**Daily calorie del. (% of requ.), mean±Std.**	73.54 (29.58)	86.69 (26.65)	58.10 (23.81)	**<0.0001**
**Daily calorie del. (% of requ.) from EN, mean±Std.**	50.63 (34.69)	61.63 (35.43)	38.25 (28.76)	**<0.0001**
**Daily calorie del. (% of requ.) from PN, mean±Std.**	14.12 (23.91)	14.18 (25.38)	14.12 (22.03)	0.9576
**Daily Calorie del. (% of requ.) from prop., mean±Std.**	8.80 (7.69)	10.88 (7.75)	5.81 (6.64)	**<0.0001**
**Daily protein del. (g/kg BW/d), mean±Std.**	0.69 (0.35)	0.82 (0.34)	0.55 (0.29)	**<0.0001**
**Daily protein del. (g/KG BW/d) from EN, mean±Std.**	0.48 (0.36)	0.61 (0.38)	0.35 (0.27)	**<0.0001**
**Daily protein del. (g/kg BW/d) from PN, mean±Std.**	0.21 (0.36)	0.21 (0.39)	0.21 (0.33)	0.7757

Abbreviations: kcal, kilocalories; del., delivery; requ., requirements; Std., standard deviation; EN, enteral nutrition; PN, parenteral nutrition; prop., propofol; g, gram; kg, kilogram; BW, actual body weight; d, day.

Supplementary Table S5: Univariable and multivariable analyses of prognostic factors for ICU survival according to adequacy of protein delivery.

Parameter	Univariable		Multivariable	
	HR (95%CI)	p-value	aHR (95%CI)	p=
**Mean protein del. ≥0.7 vs. <0.7 g/kg BW/d***	0.496 (0.250-0.983)	**0.045**	0.558 (0.264-1.178)	0.126
**Age**	1.068 (1.026-1.112)	**0.001**	1.093 (1.031-1.158)	**0.003**
**SAPSII at ECMO Start**	1.037 (1.005-1.069)	**0.023**	1.002 (0.964-1.042)	0.913
**BMI**	1.042 (0.996-1.089)	0.074	1.059 (1.006-1.115)	**0.028**

*Patients were divided in two groups according to the adequacy of protein supply (mean protein delivery ≥0.7 vs. <0.7 g/kg BW/d over the course of ECMO therapy).

Harrel's C of the overall model: 0.733 (standard error = 0.045).

Abbreviations: del., delivery; requ., requirements; vs., versus; g, gram; kg, kilogram; BW, actual body weight; d, day; SAPSII, Simplified Acute Physiology Score II; ECMO, extracorporeal membrane oxygenation; BMI, body mass index; HR, hazard ratio; aHR, adjusted hazard ratio; CI, confidence interval.

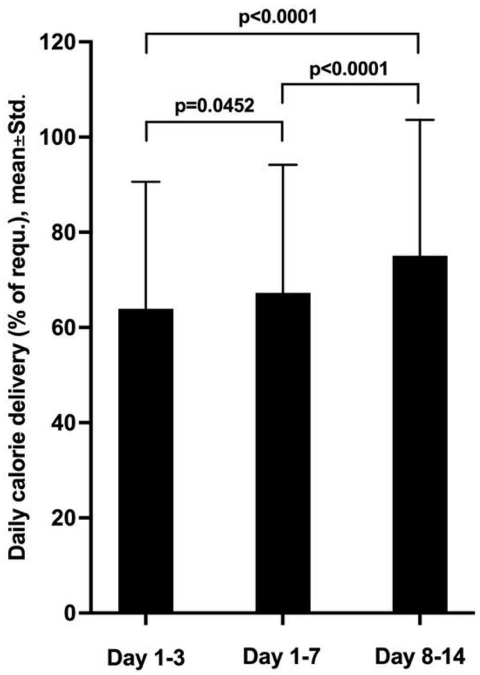

Schneeweiss-Gleixner et al; Supplementary Figure S1A

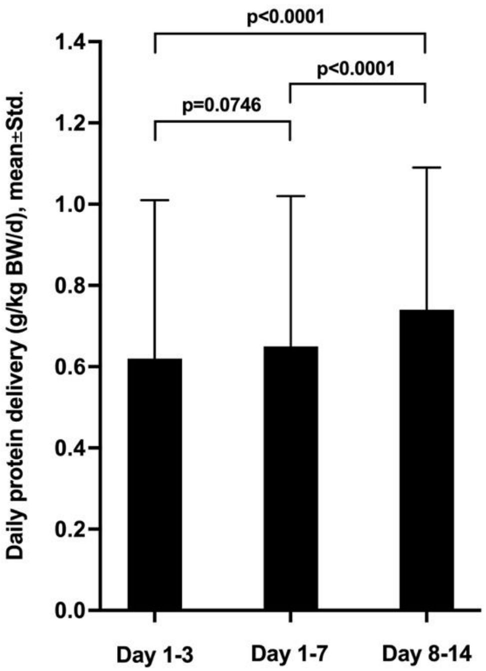

Schneeweiss-Gleixner et al; Supplementary Figure S1B

Supplemental Figure S1: Changes in nutrition support over the course of ECMO therapy.
Mean ± Standard deviation of daily calorie (Figures S1A) and protein (Figure S1B) delivery for the respective time period during ECMO support are shown.
Abbreviations: requ., requirements; Std., standard deviation; g, gram; kg, kilogram; BW, actual body weight; d, day.

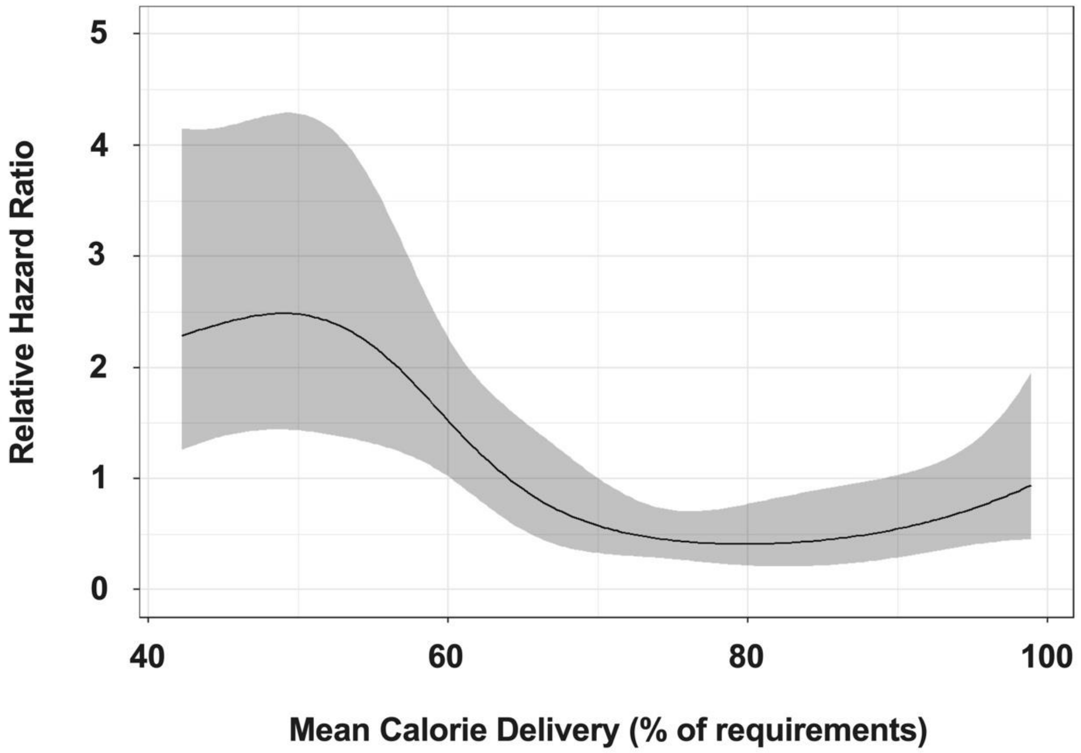

Schneeweiss-Gleixner et al; Supplementary Figure S2A

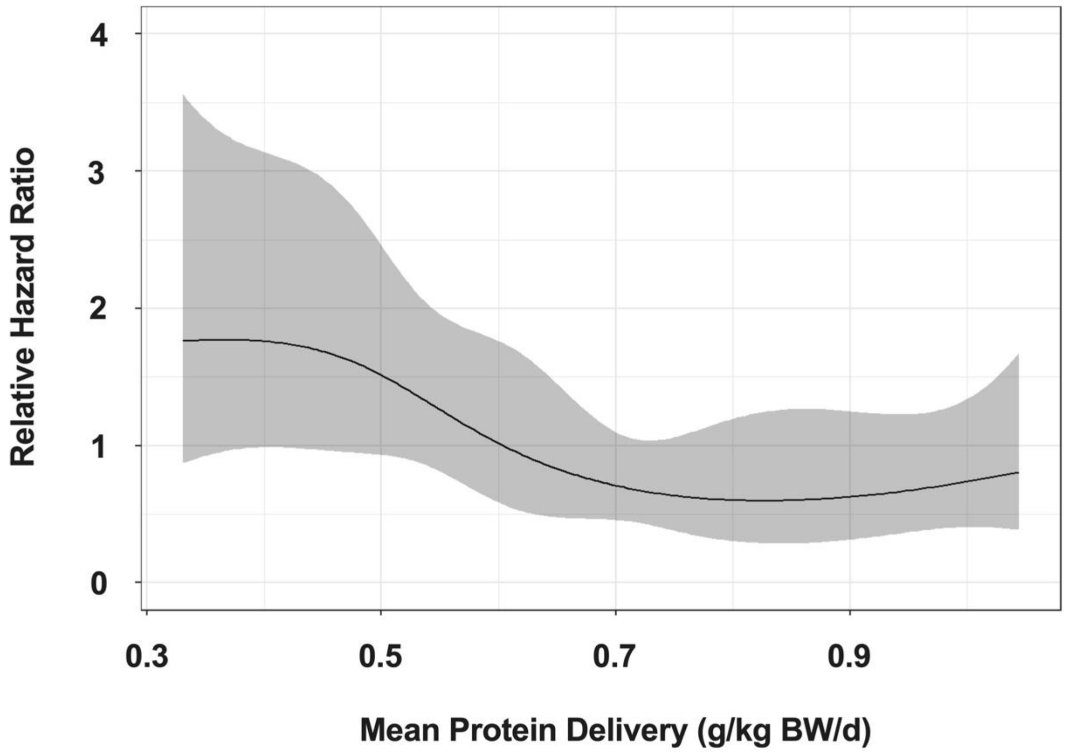

Schneeweiss-Gleixner et al; Supplementary Figure S2B

Supplementary Figure S2: Restricted cubic spline analyses for mean calory and protein delivery.

Relative hazard ratio function and 95 % pointwise confidence band estimated by a restricted cubic spline function for quantifying the effect of mean calorie (% of requirements; A) and protein (g/kg BW/d; B) delivery on ICU survival after adjustment for age, BMI, and SAPSII at ECMO start. Smaller hazard ratios indicate better survival.
